# Impact of Overweight on Response to Dupilumab Treatment in Chronic Rhinosinusitis with Nasal Polyps

**DOI:** 10.3390/nu16172982

**Published:** 2024-09-04

**Authors:** Michael Habenbacher, Ulrich Moser, Ahmed Abaira, Peter Valentin Tomazic, Peter Kiss, Clemens Holzmeister, Jakob Pock, Katharina Walla, Angelika Lang, Alexandros Andrianakis

**Affiliations:** Department of Otorhinolaryngology, Medical University of Graz, 8036 Graz, Austria

**Keywords:** obesity, overweight, dupilumab, biologic, chronic rhinosinusitis, CRSwNP

## Abstract

Chronic rhinosinusitis with nasal polyps (CRSwNP) significantly impacts quality of life and often presents therapeutic challenges, with biologics like dupilumab showing promise in managing severe, uncontrolled cases. The aim of this study was to assess the influence of overweight on the effectiveness of dupilumab in patients with uncontrolled CRSwNP. This retrospective study analyzed treatment outcomes of 75 CRSwNP patients receiving dupilumab, categorizing them into underweight/normal-weight (BMI ≤ 24.9 kg/m^2^) and overweight/obese (BMI ≥ 25 kg/m^2^) groups. Outcome measures included changes in nasal polyp score (NPS) and sinonasal outcome test (SNOT-22) scores. Results demonstrated that the underweight/normal-weight group experienced significantly greater improvements in NPS and a higher rate of total NPS improvement compared to the overweight/obese group. While SNOT-22 scores improved in both groups, no significant differences were observed. Among patients with comorbid asthma, the underweight/normal-weight subgroup also showed significantly better outcomes, including greater reductions in both NPS and SNOT-22 scores. Multiple regression analysis identified BMI as an independent prognostic factor for NPS outcomes. The findings suggest that overweight/obesity adversely affects the response to dupilumab in CRSwNP, emphasizing the need for personalized treatment strategies considering BMI.

## 1. Introduction

Chronic rhinosinusitis (CRS) affects approximately 5–12% of the global population, making it one of the most prevalent chronic conditions worldwide. A particularly severe form of this condition, namely chronic rhinosinusitis with nasal polyps (CRSwNP), is defined by bilateral inflammation of the nasal cavities/paranasal sinuses and the presence of inflammatory polypoid projections originating from the nasal mucosa [1,2]. CRSwNP constitutes about 25–30% of all CRS cases and has a prevalence ranging from 2% to 4% in Western countries [3,4]. This subtype imposes significant socioeconomic and therapeutic burdens on healthcare systems due to its high treatment costs [1,5]. Clinically, CRSwNP presents with symptoms, including nasal obstruction, reduced/absent sense of smell, anterior rhinorrhea, post-nasal drip, paranasal pressure, and facial pain. These symptoms substantially impair patients’ quality of life and contribute to considerable healthcare challenges [6,7]. CRSwNP is often associated with comorbidities such as asthma bronchiale and NSAID-exacerbated respiratory disease (N-ERD) [1,2]. Standard treatment options include topical corticosteroids (COS), systemic COS, and endoscopic sinus surgery (ESS) [1]. However, up to 40% of patients continue to experience symptoms or recurrences of nasal polyps despite standard treatment [1,8].

The main feature of CRS pathophysiology is the loss of healthy barrier function in the mucosa of the paranasal sinuses, resulting in a chronic inflammatory response that fails to resolve. Based on the molecular pathways of mucosal inflammation, three endotypes can be distinguished, namely types 1–3. CRSwNP is classified as a type 2 disease, which is the most well-described endotype and by far the predominant form in the Western world, whereas type 1 is more common in the Asian population [9]. The type 2 immune response is characterized by infiltration/accumulation of eosinophils and mast cells in the inflamed mucosa. At the beginning of this process, damage-associated molecular patterns activate innate lymphoid cells of group 2 (ILC2) and stimulate dendritic cells to promote the proliferation of T helper 2 lymphocytes (Th2). Following stimulation, ILC2 and Th2 release high amounts of interleukin-4 (IL-4), IL-5, and IL-13. These canonical cytokines are regarded as the main drivers of type 2 inflammation. Type 3 inflammation is characterized by Th17, IL-17, IL-22, and IL-23 [1,10].

Recent therapeutic advancements in uncontrolled CRSwNP have introduced biologic medications, including monoclonal antibodies targeting the key type 2 inflammatory mediators IL-4Rα, IgE, and IL-5, with established guidelines for prescription and evaluation [1,11]. Dupilumab, a fully human monoclonal antibody targeting IL-4Rα, resulted in patients with severe, uncontrolled CRSwNP in significant symptom improvement and disease control in both phase III trials, leading to its approval as the first biologic medication for CRSwNP in 2019, next to its licenses for asthma bronchiale and atopic dermatitis [12]. A recent meta-analysis of real-life studies confirmed the effectiveness of dupilumab in patients with CRSwNP, even in routine clinical practice [13].

Obesity is a rapidly growing global health issue, currently affecting over one billion people around the world [14]. Increasing evidence highlights the significant influence of obesity on CRSwNP and other Th2-driven inflammatory diseases such as asthma bronchiale or atopic dermatitis. A recent meta-analysis of six large cohort studies reported a positive association between overweight and the development of CRSwNP [15]. Further studies have indicated that higher body mass index (BMI) in individuals with CRSwNP is an independent predictor for nasal polyp recurrence [16,17]. Experimental research has demonstrated that obesity altered the traditional Th2-inflammatory response in atopic dermatitis, leading to a more severe condition marked by an inflammatory pattern with increased Th17, which is the key mediator for type 3 disease [18]. Similar results have also been reported for CRSwNP in a clinical setting, suggesting overweight/obesity CRSwNP as a unique phenotype and endotype [17]. Moreover, anti-IL-4/IL-13 treatment (i.e., dupilumab) was ineffective in obese mice, whereas it limited the disease activity in lean mice [18]. Recent clinical research has further shown that overweight affects the response to dupilumab treatment in atopic dermatitis [19,20].

To the best of our knowledge, there is no study evaluating the impact of overweight on response to biologic treatment in patients with CRSwNP in a real-life clinical setting. Therefore, in the current study, we aimed to assess the potential influence of overweight on response to dupilumab treatment in patients with severe, uncontrolled CRSwNP. We hypothesized that overweight/obese patients present a worse response to dupilumab therapy than normal/underweight patients.

## 2. Materials and Methods

### 2.1. Subjects and Study Design

This retrospective, single-arm, longitudinal cohort study was conducted at the Department of Otorhinolaryngology, Medical University of Graz, Austria. All patients diagnosed with uncontrolled CRSwNP, who were treated with dupilumab between April 2020 and September 2023 and completed the 6-month follow-up visit, were included in this study. The diagnosis of CRSwNP was based on the European Position Paper on Rhinosinusitis and Nasal Polyps (EPOS) criteria [1]. Exclusion criteria were the following: pathologies other than primary CRSwNP, insufficient recorded data, failure to follow-up, and discontinuation of dupilumab before the 6-month follow-up visit. Similar to previous studies [17,19,21], patients were divided according to their BMI into an underweight/normal-weight group (≤24.9 kg/m^2^) and overweight/obese group (≥25 kg/m^2^). Outcome of dupilumab treatment was compared between the weight groups.

### 2.2. Criteria for Dupilumab Treatment

The prescription of dupilumab for CRSwNP patients at our Department followed the EPOS 2020 indication criteria of biologics [1]: (1) presence of nasal polyps, (2) previous ESS or medical contraindication against surgery, (3) at least 3 out of 5 criteria: evidence of type 2 inflammation (blood eosinophil count (EOS) ≥ 0.25 × 10^9^/L or total serum immunoglobulin E (IgE) ≥ 100 kU/L); need of ≥2 courses systemic COS per year; sinonasal outcome test–22 (SNOT-22) score ≥ 40; impaired sense of smell (≥1 point on subitem “smelling” in SNOT-22 or hyposmia in sniffing sticks identification test 12 [SSIT-12]); comorbid asthma requiring regular inhaled corticosteroids or history of N-ERD. Dupilumab was administered s.c. at a dosage of 300 mg every two weeks. The first administration of dupilumab was monitored medically at our outpatient clinic.

### 2.3. Clinical Assessment

Patient’s clinical characteristics and medical history (age, sex, BMI, number of previous ESS, comorbidities including asthma and NERD) were recorded during the indication visit. Clinical examination was carried out before beginning biologic treatment at the indication visit (7–14 days prior to start) and six months later. Clinical examination included the following: nasal endoscopy to determine nasal polyp size, evaluation of SNOT-22 questionnaire, and blood test assessing serum IgE and EOS. Hypereosinophilia was defined as ≥1.5 × 10^9^/L. Nasal polyp size was assessed by using the well-established Meltzer Nasal Polyp Score (NPS). This is a clinical score system grading nasal polyp size (0–4 points per side, both sides summed up to total score of 0–8; 0: no polyps, 1: polyps limited to the middle meatus, 2: multiple polyps occupying the middle meatus, 3: polyps extending beyond middle meatus, 4: polyps completely obstructing the nasal cavity) [6]. The SNOT-22 is a 22-item questionnaire designed to assess how CRS affects patients’ quality of life. SNOT-22 is the recommended patient-reported outcome measure (PROM) in clinical CRS research [11,22]. It covers five domains: rhinologic symptoms, extra-nasal rhinologic symptoms, ear and facial symptoms, psychological symptoms, and sleep dysfunction. Patients evaluate their symptoms on a scale from 0 to 5. The total score ranges from 0 to 110, with higher scores reflecting greater symptom burden. Improvement of asthma symptoms in patients with comorbid asthma was assessed at the follow-up visit by patients’ subjective reports (dichotomous value: yes/no).

### 2.4. Outcome Measures

The outcome of dupilumab treatment was evaluated at the 6-month follow-up visit. The primary endpoint was the absolute change in NPS, defined as the difference between baseline NPS and 6-month follow-up NPS. Secondary endpoints were the following: reaching an NPS of 0, absolute change in SNOT-22 score (difference between baseline SNOT-22 and 6-month follow-up SNOT-22 scores), and reaching a “clinically meaningful SNOT-22 change”, defined as SNOT-22 < 40 or SNOT-22 reduction ≥ 12 points (minimal clinically important difference for medically-managed CRS [11,22]).

### 2.5. Statistics

SPSS © statistical software, version 29.0 (IBM ©, Armonk, NY, USA) was used for statistical analysis. Statistical significance level (Type I error rate = α-level) was set to a *p*-value of <0.05, two sided. Continuous variables were presented as means and standard deviations (SD). Categorical variables were depicted as absolute numbers and percentages (%). Independent *t*-test was performed to compare continuous variables between the weight groups. Paired *t*-test was performed for within-group comparisons of continuous variables. Cohen’s d was used to assess the effect size of statistically significant differences in *t*-tests. Pearson’s chi-squared test was used to compare unpaired categorical variables between groups. In cases of expected cell frequencies less than 5, the Fisher–Yates test was applied. The effect size of statistically significant differences in Fisher’s exact and Pearson’s chi-squared tests was expressed by φ-coefficient. McNemar test was performed for intra-group comparisons of dichotomous variables. In order to evaluate the prognostic capacity of BMI on NPS outcome after dupilumab treatment, a multiple linear regression analysis with a stepwise model was performed. Parameters included in the analysis were age, sex, number of prior ESS, presence of comorbid asthma, presence of N-ERD, baseline NPS, baseline SNOT-22, baseline EOS, baseline IgE, and presence of hyposmia. All the necessary conditions for a multiple linear regression analysis were met. Subgroup analysis of dupilumab treatment outcome was performed for asthma patients and patients diagnosed with N-ERD.

## 3. Results

### 3.1. Baseline Characteristics

A total of 75 CRSwNP patients (36 females [48%] and 39 males [52%]) treated with dupilumab at our academic center were included in this study. The mean age of the cohort was 51.2 years (SD 14.7). The median number of prior ESS was 2, with a range of 0–7. Two patients (2.6%) did not undergo previous ESS due to medical reasons. The mean BMI was 26.5 (SD 4.1). Of the total patients, 19% (n = 14), 44% (n = 33), and 37% (n = 28) fulfilled 3, 4, and 5 out of the 5 indication criteria, respectively. The most common criteria were impaired sense of smell (96%, n = 72), evidence of type 2 inflammation (95%, n = 71), baseline SNOT-22 score > 40 (80%, n = 60), need of ≥2 courses systemic COS per year (76%, n = 57), and comorbid asthma (66%, n = 50). Eighteen percent of patients (n = 14) had a diagnosis of N-ERD. The mean baseline serum EOS and total IgE were 0.46 × 10^9^/L (SD 0.28) and 192.5 kU/L (SD 237.5). SSIT-12 was performed in 28 patients (37%) with a mean score of 4.6 (SD 3.3). Detailed baseline characteristics according to weight groups are presented in Table 1. There were no statistically significant differences in baseline characteristics between groups (*p* > 0.05). The mean BMI in the underweight/normal weight group and overweight/obese group were 22.6 (SD 1.7) and 29.1 (SD 3.1), respectively.

### 3.2. Dupilumab Treatment Outcome of the Total Cohort

In the total cohort, the NPS decreased statistically significantly by 3.7 (SD 2.1) from baseline 4.8 (SD 1.8) to 1.1 (SD 1.4) [t(74) = 15.3, *p* ≤ 0.001, d = 1.7]. Thirty-nine patients (52%) showed an NPS of 0 [McNemar(75) = 33; *p* ≤ 0.001] at the follow-up visit. The SNOT-22 improved statistically significantly by 30.8 (SD 21.1) from baseline 56.1 (SD 20.7) to 25.3 (SD 19.6) [t(74) = 12, *p* ≤ 0.001, d = 1.4]. Overall, 69 patients (92%) achieved a SNOT-22 score of <40 or declined at least 12 points.

### 3.3. Dupilumab Treatment Outcome According to Weight Groups

There were no statistically significant differences in baseline outcome parameters between weight groups (*p* > 0.05); see Table 2. A statistically significant difference was found in the primary endpoint, “Change in NPS”, between the weight groups [t(73 = 2.98, *p* = 0.004, d = 0.7]. Patients in the underweight/normal weight group had a 1.4 [CI(0.47, 2.36] higher reduction in NPS than patients in the overweight/obese weight group. Moreover, under/normal weight was associated with a significantly higher rate of reaching an NPS of 0 [77% vs. 29%; χ^2^(1) = 16.46, *p* < 0.001, φ = 0.46]. There were no statistically significant differences in change in SNOT-22 score [t(73 = 1.64, *p* = 0.104] or clinically meaningful SNOT-22 changes between groups [97% vs. 89%; χ^2^(1) = 1.47, *p* = 0.3921]. Out of the 50 patients with comorbid asthma, 48 patients (96%) reported an improvement in asthma symptoms. Detailed results are presented in Table 2.

### 3.4. Weight Subgroups Analysis of Dupilumab Treatment Outcome

#### 3.4.1. N-ERD Weight Subgroups

Out of 14 patients diagnosed with N-ERD, 6 patients were underweight/normal weight, while the remaining 8 individuals were overweight/obese. There were no statistically significant differences in baseline NPS and SNOT-22 scores (*p* > 0.05). Regarding treatment outcomes, underweight/normal weight individuals had a statistically significant higher change in NPS (*p* = 0.047), lower follow-up NPS (*p* = 0.014), higher rate of reaching an NPS of 0 (*p* = 0.026), higher reduction in SNOT-22 (*p* = 0.025) and lower follow-up SNOT-22 (*p* = 0.030). Detailed results are shown in Table 3.

#### 3.4.2. Asthma Weight Subgroups

Of the 50 patients with comorbid asthma, 22 were classified as underweight/normal weight, while the other 28 were categorized as overweight/obese. No statistically significant differences were observed in the baseline NPS and SNOT-22 scores in asthma patients between weight subgroups (*p* > 0.05). In terms of treatment outcomes, individuals in the underweight/normal-weight group demonstrated a significantly greater reduction in NPS (*p* = 0.014), lower NPS at follow-up (*p* = 0.011), a higher likelihood of achieving an NPS of 0 (*p* < 0.001), a greater reduction in SNOT-22 scores (*p* = 0.026), and lower SNOT-22 scores at follow-up (*p* = 0.037). Detailed results are presented in Table 4.

### 3.5. BMI as Independent Prognostic Factor for NPS Outcome after Dupilumab Treatment

Multiple regression analysis identified BMI and age as independent prognostic factors of NPS outcome after dupilumab treatment. BMI had the highest impact on NPS outcome, followed by age; see Table 5. Sex, number of prior ESS, presence of comorbid asthma, presence of N-ERD, baseline NPS, baseline SNOT-22, baseline EOS, baseline IgE, and presence of hyposmia had no significant independent impact on NPS outcome after dupilumab treatment (*p* > 0.05).

### 3.6. Side Effects and Blood Parameter Outcomes

A minority of patients (10%, n = 8) presented hypereosinophilia as a dupilumab-induced side effect at the follow-up visit. No other medication-related side effects were observed. The mean baseline serum EOS and total IgE were 0.66 *×* 10^9^/L (SD 0.51) and 84.8 kU/L (SD 112.3). There were no statistically significant differences in serum EOS and IgE values or hypereosinophilia rates between the weight groups (*p* > 0.05). Detailed data regarding blood parameters are presented in Table 6.

## 4. Discussion

To the best of our knowledge, this is the first study investigating the impact of overweight on the response to dupilumab treatment in patients with uncontrolled CRSwNP. The results revealed that overweight and obese patients exhibit a significantly reduced response to dupilumab therapy compared to their normal-weight counterparts. This aligns with previous research indicating that higher BMI is a predictor of poorer outcomes of biologic therapy in type 2 inflammatory diseases [19,20].

Since its approval in 2019, dupilumab has broadened the range of treatment options for patients suffering from uncontrolled CRSwNP. Several real-life studies have confirmed the effectiveness of dupilumab in the treatment of uncontrolled CRSwNP [13]. The treatment outcomes in our total cohort are consistent with prior clinical trials [12] and real-world data [13] on patient responses to dupilumab after six months of treatment, especially regarding direct objective and subjective measures of nasal function such as NPS and SNOT-22. These results confirm that our cohort reflects the typical patient population benefiting from dupilumab therapy as reported in the literature.

Evidence of the detrimental effects of overweight on type 2-driven inflammatory diseases is growing. In an experimental mouse model of atopic dermatitis, Bapat et al. [18] observed that obesity alters the conventional Th2-dominant profile, exacerbating the condition with pronounced Th17-driven inflammation. Due to this irregular Th17-predominant inflammation in obese mice with a usually Th2-driven disease, the researchers further administered anti-IL-4/IL-13 antibodies (e.g., dupilumab) to lean and obese mice. The antibody treatment was not only ineffective in obese mice but also exacerbated the conditions’ activity compared to lean mice. Recently, similar results were also reported for CRSwNP in a clinical trial. The authors characterized the inflammatory pattern in nasal polyp tissue of 325 CRSwNP patients undergoing ESS by immunohistochemical staining. They found that overweight/obese individuals exhibit an amplified Th2/Th17 co-existing inflammatory profile compared to underweight and normal weight controls, suggesting overweight/obese CRSwNP as a unique endotype and phenotype [17]. However, this study included Asian patients, and type 3 inflammation is more common in Asian countries [9]. Future studies involving individuals from western countries are warranted to further validate these results.

The interplay between obesity and asthma is intricate and involves various physiological and inflammatory mechanisms. Obesity exacerbates asthma by promoting a state of chronic inflammation and reducing lung function, which in turn increases the frequency and intensity of asthma symptoms. This is due to the presence of excess adipose tissue in obese individuals, which produces higher levels of inflammatory cytokines such as leptin, TNF-α, and IL-6. These cytokines contribute to increased airway inflammation and hyperresponsiveness. Additionally, obesity-related mechanical factors, like decreased lung volumes and increased airway resistance, further impair respiratory function. The impact of obesity on the effectiveness of biologic treatments like dupilumab is also noteworthy. Biologics targeting the IL-4, IL-5, and IL-13 pathways are generally effective in reducing asthma exacerbations and improving lung function. However, their efficacy can be affected by the patient’s BMI. Obese individuals often exhibit a combined inflammatory response involving both Th2 and Th17 pathways, which may reduce the effectiveness of Th2-targeted therapies such as dupilumab [23,24,25].

Another aspect to consider in this context is the potential modifications of pharmacokinetics induced by obesity. Pharmacokinetics impact the amount of active drug that reaches its target intact, thereby impacting its effectiveness. Unlike conventional medications, dupilumab can only be administered intravenously or, as occurs in most cases, subcutaneously. Subcutaneous absorption may be hindered by presystemic elimination due to the action of soluble peptidases, endothelial endocytosis, lysosomal degradation, and interactions with phagocytic immune cells in the lymph nodes. Additionally, for monoclonal antibodies to enter the systemic circulation, they must undergo convective transport through the interstitial space into the lymphatic system, a process that can be more challenging in obese patients [26,27,28].

Recent clinical studies have demonstrated that overweight negatively influences the effectiveness of dupilumab therapy in treating atopic dermatitis [19,20]. The current study was the first to investigate the impact of overweight on the response to dupilumab treatment in patients with uncontrolled CRSwNP. A comparison of the patient’s clinical characteristics revealed no significant differences between the weight groups. Thus, the groups were adequately comparable for further statistical analysis of treatment outcome. We found that underweight/normal weight individuals had a statistically significant reduction in NPS, quantifying 1.4 on average compared to overweight/obese patients. However, statistically significant does not automatically mean clinically significant. An NPS reduction of at least 1.0 is widely accepted as clinically meaningful in CRSwNP trials, as recommended by guidelines and expert boards [11,22]. Our observed difference in change of NPS exceeded this value; hence, our findings can also be interpreted as clinically significant. Moreover, underweight/normal individuals achieved a total NPS improvement significantly more often than overweight/obese patients (48%). On the other hand, we failed to find a statistically significant difference in subjective parameters, e.g., absolute SNOT-22 change and clinically meaningful SNOT-22 changes between the weight groups. Notably, in both SNOT-22 parameters, overweight/obese people presented worse values. However, despite the extensive data validating SNOT-22’s reliability and accuracy, patient responses to PROMs may be impacted by numerous individual factors that remain largely unexplored and/or undocumented, such as anxiety/depression, interactions with healthcare providers, and clinical settings [29,30]. The discrepancy in statistically significant differences concerning NPS and SNOT-22 may be attributed to the multifaceted nature of SNOT-22, which includes various aspects of quality of life and symptomatology beyond nasal polyp burden alone. Further research is needed to understand the factors influencing these divergent outcomes. Lastly, multiple regression analysis identified BMI as an independent prognostic factor for response to dupilumab treatment. This finding enhanced the internal validity of our group comparison results and underscores the importance of considering BMI as a potential prognostic factor in the biologic management of CRSwNP.

We identified hypereosinophilia as a potential side effect of dupilumab, which aligns with findings from other research indicating an increased prevalence of elevated blood eosinophil counts in patients undergoing dupilumab therapy [31,32]. However, our cases of hypereosinophilia did not lead to hypereosinophilic syndrome or other significant clinical complications. Kemp et al. [31] and De Corso et al. [32] reported similar increases in eosinophil counts that typically resolved without the need for additional intervention. Our data did not show a significant difference in the incidence of blood eosinophilia between the underweight/normal-weight and overweight/obese groups, indicating that BMI does not appear to be a risk factor for this side effect. These findings reinforce the safety profile of dupilumab and suggest that regular monitoring of eosinophil levels is a prudent practice during treatment.

Our analysis revealed that age was also a significant, although modest, prognostic factor in response to dupilumab treatment, with older patients showing less pronounced improvements in NPS. This may be attributed to age-related changes in immune function, e.g., immunosenescence, and alterations in tissue remodeling processes. However, the available research on biologic therapy for Th2-driven diseases in the elderly is very limited [33,34]. Future studies are needed to clarify these issues.

Novel approaches for precision immunological treatment are essential to address the altered pathology caused by overweight and obesity. Recent research suggested that the downregulation of PPAR-γ, a transcription factor recently identified as crucial for the regulation of transcriptional networks in Th2 cells, may contribute to the shifted Th17-driven inflammation in obese type-2 inflammatory diseases. Rosiglitazone, a PPAR-γ agonist, decreased the intensity of the disease and reestablished the effectiveness of anti-IL-4/IL-13 treatment (e.g., dupilumab) in an experimental model [18]. Another research group discovered glucagon-like peptide 1 receptors (GLP-1) in airway epithelial cells in mice. They further observed that a GLP-1 receptor agonist reduced neutrophilic airway inflammation by deactivating ILC-2, a key mediator in CRSwNP pathophysiology, in obese mice [35,36].

While the study provides valuable contributions to the field of CRSwNP, there are some limitations that should be acknowledged. First, the relatively small sample size and single-center setting may limit the generalizability of our findings to broader populations. Second, the retrospective, observational nature of the study might introduce selection bias and uncontrolled confounding variables. Third, the lack of consistent administration of smell tests across all patients in our department prevents a comprehensive assessment of smell improvement, which is a critical symptom of CRSwNP. Fourth, we were unable to evaluate objective asthma outcomes such as FEV1 or validated asthma PROMs like the asthma control test. However, it must also be emphasized that the patients in our study were treated with dupilumab primarily due to uncontrolled sinonasal symptoms and that asthma symptoms were not the predominant patient concern. Finally, the follow-up duration of six months may not capture the long-term effects of dupilumab therapy or the potential impact of BMI changes over time on treatment outcomes. It is important to note that many patients complete their subsequent follow-ups in outpatient care, which limited our ability to provide long-term data beyond the initial six-month period. However, the vast majority of CRSwNP patients treated with dupilumab reach over 80% of NPS and symptoms reduction within the first 8–12 weeks [22].

Despite these limitations, our study provides novel real-world evidence on the impact of BMI on dupilumab treatment outcomes in patients with uncontrolled CRSwNP. Future prospective studies with larger sample sizes and longer follow-up durations are warranted to validate our findings and elucidate further the underlying mechanisms driving the association between BMI and response to biologic therapy in CRSwNP.

## 5. Conclusions

In conclusion, our study provides novel insights into the influence of overweight/obesity on the response to biologic therapy in CRSwNP. We have demonstrated that overweight/obese patients exhibit a less favorable response to dupilumab compared to their underweight/normal-weight counterparts. These findings underscore the importance of considering BMI as a potential prognostic factor in the management of CRSwNP, particularly when employing biologic therapies targeting type 2 inflammation.

## Figures and Tables

**Table 1:** Patient baseline characteristics stratified by weight groups.

Characteristic	Underweight/Normal Weight Group (n = 30)	Overweight/Obese Group (n = 45)	*p*-Value
Sex, f/m	16/14	20/25	0.487
Age in years	49.9 (SD 16.7)	51.9 (13.3)	0.570
Prior ESS	2.7 (SD 1.7)	2.2 (SD 1.4)	0.257
n° (%) of indication criteria, 3/4/5	9 (30%)/10 (33%)/11 (37%)	5 (11%)/23 (51%)/17 (38%)	0.101
Evidence of type 2 inflammation	27 (90%)	44 (98%)	0.295
Need of ≥2 courses systemic COS per year	22 (73%)	35 (78%)	0.784
Impaired sense of smell	28 (93%)	44 (97%)	0.560
Comorbid asthma	22 (73%)	28 (62%)	0.454
N-ERD	6 (20%)	8 (18%)	0.809
Baseline sEOS × 10^9^/L	0.43 (SD 0.2)	0.48 (SD 2.1)	0.485
Baseline total IgE × kU/L	136.5 (SD 127.5)	229.7 (SD 283.9)	0.096

Continuous variables are presented as means and standard deviations (SD). Categorical variables are presented as absolute numbers and percentages (%). f: female. m: male. ESS: endoscopic sinus surgery. COS: corticosteroids. N-ERD: NSAID-exacerbated respiratory disease. sEOS: serum eosinophils.

**Table 2:** Weight group comparison in dupilumab treatment outcome.

Parameter	Underweight/Normal Weight Group (n = 30)	Overweight/Obese Group (n = 45)	*p*-Value	Effect Size
Baseline NPS	5.1 (SD 1.6)	4.7 (SD 1.7)	0.323	-
Follow-up NPS	0.5 (SD 1.2)	1.5 (SD 1.4)	0.002	d = 0.75
Change in NPS	−4.6 (SD 2)	−3.2 (SD 1.9)	0.004	d = 0.38
Reaching NPS of 0	23 (77%)	13 (29%)	<0.001	φ = 0.46
Baseline SNOT-22	52.6 (SD 20.3)	58.5 (SD 20.9)	0.231	-
Follow-up SNOT-22	16.7 (SD 15.9)	31.1 (SD19.8)	0.001	d = 0.78
Change in SNOT-22	−35.9 (SD 22.3)	−27.4 (SD 21.6)	0.104	-
Clinically meaningful SNOT-22 change	29 (97%)	40 (89%)	0.392	-

Continuous variables are presented as means and standard deviations (SD). Categorical variables are presented as absolute numbers and percentages (%). NPS: nasal polyp score. SNOT-22: sinonasal outcome test 22. Clinically meaningful SNOT-22 change: achieving a SNOT-22 score < 40 or reduction ≥ 12 points. d: effect size for statistically significant differences in *t*-tests. φ: effect size of statistically significant differences in chi-squared/Fisher’s exact test.

**Table 3:** N-ERD weight subgroups comparison (N = 14).

Parameter	Underweight/Normal Weight Group (n = 6)	Overweight/Obese Group (n = 8)	*p*-Value	Effect Size
Baseline NPS	5.1 (SD 1.9)	4.4 (SD 2.4)	0.527	-
Follow-up NPS	0.1 (SD 0.4)	1.5 (SD 1.1)	0.014	d = 1.55
Change in NPS	−5 (SD 2)	−2.8 (SD 2.3)	0.047	d = 0.95
Reaching NPS of 0	5 (83%)	1 (12%)	0.026	φ = 0.71
Baseline SNOT-22	58 (SD 23.2)	54.8 (SD 12.5)	0.751	-
Follow-up SNOT-22	6 (SD 5.4)	30 (SD 23.5)	0.030	d = 1.32
Change in SNOT-22	−52 (SD 21.6)	−24.6 (SD 24.3)	0.025	d = 1.17
Clinically meaningful SNOT-22 change	6 (100%)	7 (87%)	0.571	-

Continuous variables are presented as means and standard deviations (SD). Categorical variables are presented as absolute numbers and percentages (%). NPS: nasal polyp score. SNOT-22: sinonasal outcome test 22. Clinically meaningful SNOT-22 change: achieving a SNOT-22 score < 40 or reduction ≥ 12 points. d: effect size for statistically significant differences in *t*-tests. φ: effect size of statistically significant differences in chi-squared/Fisher’s exact test.

**Table 4:** Asthma weight subgroups comparison (N = 50).

Parameter	Underweight/Normal Weight Group (n = 22)	Overweight/Obese Group (n = 28)	*p*-Value	Effect Size
Baseline NPS	4.9 (SD 1.8)	4.5 (SD 1.4)	0.410	-
Follow-up NPS	0.5 (SD 1.3)	1.4 (SD 1)	0.011	d = 0.749
Change in NPS	−4.5 (SD 2.1)	−3.1 (SD 1.4)	0.014	d = 0.728
Reaching a NPS of 0	17 (77%)	7 (25%)	<0.001	φ = 0.519
Baseline SNOT-22	53.5 (SD 18.5)	55.1 (SD 19.8)	0.783	-
Follow-up SNOT-22	18.5 (SD 17.1)	31 (SD 22.7)	0.037	d = 0.610
Change in SNOT-22	−35 (SD 19.9)	−24 (SD 18.7)	0.026	d = 0.570
Clinically meaningful SNOT-22 change	21 (95%)	25 (89%)	0.621	-
Subjective asthma-symptoms improvement	22 (100%)	26 (92%)	0.497	

Continuous variables are presented as means and standard deviations (SD). Categorical variables are presented as absolute numbers and percentages (%). NPS: nasal polyp score. SNOT-22: sinonasal outcome test 22. Clinically meaningful SNOT-22 change: achieving a SNOT-22 score < 40 or reduction ≥ 12 points. d: effect size for statistically significant differences in *t*-tests. φ: effect size of statistically significant differences in chi-squared/Fisher’s exact test.

**Table 5:** Independent prognostic factors in multiple regression analysis on NPS outcome after dupilumab treatment.

Parameter	Coefficient B	Standard Error	Standardized Beta	T	*p*-Value
BMI	0.088	0.037	0.263	2.371	0.020
Age	−0.023	0.011	−0.236	−2.141	0.036

**Table 6:** Blood parameters comparison between weight groups.

Parameter	Underweight/Normal Weight Group (n = 30)	Overweight/Obese Group (n = 45)	*p*-Value
Baseline sEOS	0.43 (SD 0.2)	0. 48 (SD 2.1)	0.485
Follow-up sEOS	0.59 (SD 0.4)	0.70 (SD 0.5)	0.370
Hypereosinophilia	1 (3%)	7 (15%)	0.134
Baseline total IgE	136.5 (SD 127.5)	229.7 (SD 283.9)	0.096
Follow-up total IgE	62.2 (SD 66.3)	99.9 (SD 133.1)	0.155

Continuous variables are presented as means and standard deviations (SD). Categorical variables are presented as absolute numbers and percentages (%). sEOS: serum eosinophil count. sEOS values are given in 10^9^/L. IgE: immunoglobuline E. IgE values are given in kU/L.

## Data Availability

The original contributions presented in the study are included in the article; further inquiries can be directed to the corresponding author.

## References

[1] Fokkens W.J., Lund V.J., Hopkins C., Hellings P.W., Kern R., Reitsma S., Toppila-Salmi S., Bernal-Sprekelsen M., Mullol J., Alobid I. (2020). European position paper on rhinosinusitis and nasal polyps 2020. Rhinology.

[2] Stevens W.W., Schleimer R.P., Kern R.C. (2017). Chronic rhinosinusitis with nasal polyps. J. Allergy Clin. Immunol. Pract..

[3] Chaaban M.R., Walsh E.M., Woodworth B.A. (2013). Epidemiology and differential diagnosis of nasal polyps. Am. J. Rhinol. Allergy.

[4] Johansson L., Akerlund A., Holmberg K., Melen I., Bende M. (2003). Prevalence of nasal polyps in adults: The Skovde population-based study. Ann. Otol. Rhinol. Laryngol..

[5] Bhattacharyya N., Villeneuve S., Joish V.N., Amand C., Mannent L., Amin N., Rowe P., Maroni J., Eckert L., Yang T. (2019). Cost burden and resource utilization in patients with chronic rhinosinusitis and nasal polyps. Laryngoscope.

[6] Bachert C., Bhattacharyya N., Desrosiers M., Khan A.H. (2021). Burden of disease in chronic rhinosinusitis with nasal polyps. J. Asthma Allergy.

[7] Liu D.T., Bartosik T.J., Campion N.J., Bayer K., Tu A., Victoria S., Besser G., Mueller C.A., Gangl K., Eckl-Dorna J. (2022). Chronic rhinosinusitis symptoms differentially impact the likelihood of major depressive disorders. Laryngoscope Investig. Otolaryngol..

[8] DeConde A.S., Mace J.C., Levy J.M., Rudmik L., Alt J.A., Smith T.L. (2017). Prevalence of polyp recurrence after endoscopic sinus surgery for chronic rhinosinusitis with nasal polyposis. Laryngoscope.

[9] Yao Y., Zeng M., Liu Z. (2022). Revisiting Asian chronic rhinosinusitis in the era of type 2 biologics. Clin. Exp. Allergy.

[10] Czerwaty K., Piszczatowska K., Brzost J., Ludwig N., Szczepański M.J., Dżaman K. (2022). Immunological Aspects of Chronic Rhinosinusitis. Diagnostics.

[11] Fokkens W.J., Viskens A.S., Backer V., Conti D., De Corso E., Gevaert P., Scadding G.K., Wagemann M., Bernal-Sprekelsen M., Chaker A. (2023). EPOS/EUFOREA update on indication and evaluation of Biologics in Chronic Rhinosinusitis with Nasal Polyps 2023. Rhinology.

[12] Bachert C., Han J.K., Desrosiers M., Hellings P.W., Amin N., Lee S.E., Mullol J., Greos L.S., Bosso J.V., Laidlaw T.M. (2019). Efficacy and safety of dupilumab in patients with severe chronic rhinosinusitis with nasal polyps (LIBERTY NP SINUS-24 and LIBERTY NP SINUS-52): Results from two multicentre, randomised, double-blind, placebo-controlled, parallel-group phase 3 trials. Lancet.

[13] Reale M., Licci G., Orlando P., Matucci A., Trabalzini F., Maggiore G., Gallo O. (2024). Efficacy and safety of dupilumab in the treatment of CRSwNP in the real-life setting: A review of the literature. Eur. Arch. Otorhinolaryngol..

[14] NCD Risk Factor Collaboration (NCD-RisC) (2017). Worldwide trends in body-mass index, underweight, overweight, and obesity from 1975 to 2016: A pooled analysis of 2416 population-based measurement studies in 128·9 million children, adolescents, and adults. Lancet.

[15] Hoying D., Miller K., Tanzo J., Kim J., Bena J., Burguera B., Chaaban M.R. (2024). Evaluating the Association of Obesity and Chronic Rhinosinusitis: A Systematic Review and Meta-analysis. Otolaryngol. Head Neck Surg..

[16] Xie S., Jiang S., Fan R., Gao K., Shui J., Wang F., Xie Z., Zhang H., Jiang W. (2023). Elevated body mass index increased the risk of recurrence in Chinese patients with chronic rhinosinusitis. Am. J. Otolaryngol..

[17] Chen W., Bai Y., Fang P., Chen J., Wang X., Li Y., Luo X., Xiao Z., Iyer R., Shan F. (2024). Body mass index’s effect on CRSwNP extends to pathological endotype and recurrence. Rhinology.

[18] Bapat S.P., Whitty C., Mowery C.T., Liang Y., Yoo A., Jiang Z., Peters M.C., Zhang L.-J., Vogel I., Zhou C. (2022). Obesity alters pathology and treatment response in inflammatory disease. Nature.

[19] Gu C., Wu Y., Luo Y., Wang S., Yin H., Gao Y., Wang C., Yao X., Li W. (2022). Real-world efficacy and safety of dupilumab in Chinese patients with atopic dermatitis: A single-centre, prospective, open-label study. J. Eur. Acad. Dermatol. Venereol..

[20] Wang A., Zhou Y., Luo Y., Gao Y., Chen J., Li W., Luo X., Yao X. (2023). High loading-dose of dupilumab resulted in rapid disease control in pediatric patients with atopic dermatitis. Front. Immunol..

[21] Zhang Y., Shen S., Liu Y., Wang Z., Wang Q., Li Y., Wang C., Lan F., Zhang L. (2022). The influence of body mass index on glucocorticoid insensitivity in chronic rhinosinusitis with nasal polyps. J. Pers. Med..

[22] Bachert C., Han J.K., Wagenmann M., Hosemann W., Lee S.E., Backer V., Mullol J., Gevaert P., Klimek L., Prokopakis E. (2021). EUFOREA expert board meeting on uncontrolled severe chronic rhinosinusitis with nasal polyps (CRSwNP) and biologics: Definitions and management. J. Allergy Clin. Immunol..

[23] Listyoko A.S., Okazaki R., Harada T., Inui G., Yamasaki A. (2024). Impact of obesity on airway remodeling in asthma: Pathophysiological insights and clinical implications. Front. Allergy.

[24] Tashiro H., Kurihara Y., Kuwahara Y., Takahashi K. (2024). Impact of obesity in asthma: Possible future therapies. Allergol. Int..

[25] Garg D., Que L.G., Ingram J.L. (2024). Effects of biological therapies on patients with Type-2 high asthma and comorbid obesity. Front. Pharmacol..

[26] Matucci A., Micheletto C., Vultaggio A. (2023). Severe Asthma and Biologics: Managing Complex Patients. J. Investig. Allergol. Clin. Immunol..

[27] Ryman J.T., Meibohm B. (2017). Pharmacokinetics of Monoclonal Antibodies. CPT Pharmacomet. Syst. Pharmacol..

[28] Matera M.G., Calzetta L., Rogliani P., Cazzola M. (2019). Monoclonal antibodies for severe asthma: Pharmacokinetic profiles. Respir. Med..

[29] Yang N., Waytz A., Soler Z.M., Overdevest J.B., Gudis D.A. (2024). The effects of priming on rhinologic patient reported outcome measures: A randomized controlled trial. Rhinology.

[30] Phillips K.M., Sedaghat A.R. (2022). Depression and Anxiety: Considerations for Interpretation of the SNOT-22 (22-Item Sinonasal Outcome Test). Otolaryngol. Head Neck Surg..

[31] Kemp P., van der Lans R.J.L., Otten J.J., GFJPM A., Benoist L.B.L., Cornet M.E., Hoven D.R., Rinia B., Verkest V., Fokkens W.J. (2024). Hypereosinophilia during dupilumab treatment in patients with chronic rhinosinusitis with nasal polyps. Rhinology.

[32] De Corso E., Montuori C., Baroni S., Mastrapasqua R.F., Porru D.P., D’auria L.M., D’agostino G., Penazzi D., De Maio G., Onori M.E. (2024). Temporal trends of blood eosinophilia in severe uncontrolled CRSwNP treated with dupilumab: A real-life study. Eur. Arch. Otorhinolaryngol..

[33] Ji H., Tan L.D., Hafzalla G.W., Nguyen N., Alismail A. (2024). Navigating biologic therapies in elderly asthma. Respir. Med..

[34] Benfante A., Principe S., Battaglia S., Scichilone N. (2019). Are biological drugs effective and safe in older severe asthmatics?. Expert Opin. Drug Saf..

[35] Toki S., Goleniewska K., Reiss S., Zhang J., Bloodworth M.H., Stier M.T., Zhou W., Newcomb D.C., Ware L.B., Stanwood G.D. (2018). Glucagon-like peptide 1 signaling inhibits allergen-induced lung IL-33 release and reduces group 2 innate lymphoid cell cytokine production in vivo. J. Allergy Clin. Immunol..

[36] Toki S., Newcomb D.C., Printz R.L., Cahill K.N., Boyd K.L., Niswender K.D., Peebles R.S. (2021). Glucagon-like peptide-1 receptor agonist inhibits aeroallergen-induced activation of ILC2 and neutrophilic airway inflammation in obese mice. Allergy.

